# Type V Secretion Systems: An Overview of Passenger Domain Functions

**DOI:** 10.3389/fmicb.2019.01163

**Published:** 2019-05-31

**Authors:** Ina Meuskens, Athanasios Saragliadis, Jack C. Leo, Dirk Linke

**Affiliations:** Department of Biosciences, Section for Genetics and Evolutionary Biology, University of Oslo, Oslo, Norway

**Keywords:** secretion systems, AT, virulence, bacterial outer membrane, Gram-negative microorganisms

## Abstract

Bacteria secrete proteins for different purposes such as communication, virulence functions, adhesion to surfaces, nutrient acquisition, or growth inhibition of competing bacteria. For secretion of proteins, Gram-negative bacteria have evolved different secretion systems, classified as secretion systems I through IX to date. While some of these systems consist of multiple proteins building a complex spanning the cell envelope, the type V secretion system, the subject of this review, is rather minimal. Proteins of the Type V secretion system are often called autotransporters (ATs). In the simplest case, a type V secretion system consists of only one polypeptide chain with a β-barrel translocator domain in the membrane, and an extracellular passenger or effector region. Depending on the exact domain architecture of the protein, type V secretion systems can be further separated into sub-groups termed type Va through e, and possibly another recently identified subtype termed Vf. While this classification works well when it comes to the architecture of the proteins, this is not the case for the function(s) of the secreted passenger. In this review, we will give an overview of the functions of the passengers of the different AT classes, shedding more light on the variety of functions carried out by type V secretion systems.

## Introduction

Bacteria in general display a great variety of proteins on their cell surface, serving functions in nutrient transport, signaling, adhesion, or virulence. The proteins and protein complexes responsible for secretion in Gram-negative bacteria can be divided into categories, termed type I through type IX secretion systems (Costa et al., 2015; Green and Mecsas, 2016). These secretion systems differ in their complexity, with some of them consisting of only one polypeptide chain, like some of the type V secretion systems, to very intricate machineries consisting of multiple proteins building a complex, sometimes spanning several membranes. Compared with multi-subunit secretion systems, type V secretion systems seem somewhat peculiar. In comparison to most of the other secretion systems, they are much smaller, and only span the Gram-negative outer membrane (OM) (Leo et al., 2012). Type V secretion systems have no obvious energy source for transport, as there is no chemical energy such as ATP available in the periplasm, and no stable proton or other ion gradients exist across the OM. This has led to the name “autotransporter” (AT) that suggests a completely self-sufficient system for secretion (Klauser et al., 1993). Today we know of multiple factors that are involved in the secretion of ATs, but the source of energy for the secretion is still a matter of debate (Thanassi et al., 2005; Kang’ethe and Bernstein, 2013; Drobnak et al., 2015; Oberhettinger et al., 2015).

**FIGURE 1:**
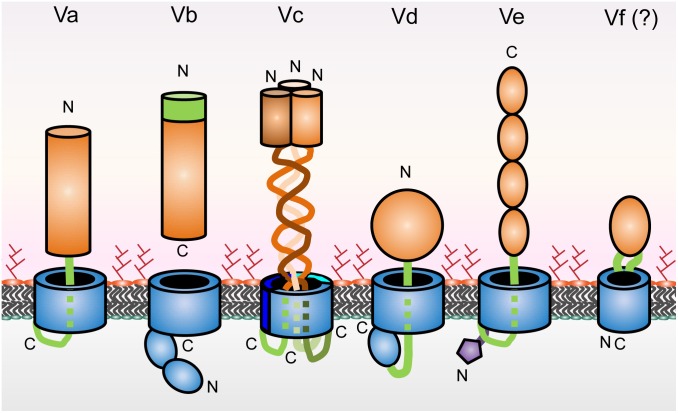
Schematic of type V secretion system subclasses. β-barrels and POTRA domains are shown in blue, linkers and TPS domains in green, and passengers in orange. The periplasmic extension of type Ve proteins is in purple. The positions of the N- and C-termini are indicated. Type Vf is not fully established as part of the type V secretion scheme; this is denoted by the question mark.

Type V secretion systems come in different forms depending on their structural features and domain organization (Figure 1). Type V ATs are therefore divided into sub-classes, type Va through type Ve, and possibly the very recently suggested type Vf (Grijpstra et al., 2013). While sub-classification according to the domain structure of ATs is useful to show differences in general principles of their organization and biogenesis, this does not usually reflect the secreted passengers’ function(s). AT passengers function in very diverse ways, ranging from adhesins or enzymes to toxic proteins. Table 1 gives a general overview of functionalities of passengers from the different subclasses. While many reviews concentrate on the topology and biogenesis of ATs, we deliberately focus on the functions of the passenger domains of ATs. We also give a short overview of the different topologies of AT sub-classes and their biogenesis.

**Table 1:** Functions of type V secretion system passenger domains.

Function	Mode of action Example				AT-Type	References
Enzyme	Protease	SPATE	Group I	EspC (*Escherichia coli*)	Va	Stein et al., 1996
				EspP (*Escherichia coli*)		Roman-Hernandez et al., 2014
			
			Group II	Hbp (*Escherichia coli*)	Va	Soprova et al., 2010
				Pic (*Escherichia coli*)		Abreu et al., 2016
		
		SPATE-like	IgA protease (*Neisseria gonorrhoeae*)		Va	Hulks and Plaut, 1978; Diebel et al., 2004
		
		Non-SPATE	NalP (*Neisseria meningitidis*)		Va	Arenas et al., 2013
			Ssph1/2 (*Serratia marcescens*)			Ohnishi et al., 1997; Kida et al., 2008
			LepA (*Pseudomonas aeruginosa*)		Vb	Ohnishi and Horinouchi, 2009
	
	Lipase/esterase	EstA (*Pseudomonas aeruginosa*)			Va	Wilhelm et al., 2007
		McaP (*Moraxella catarrhalis*)				Lipski et al., 2007
		EstA (*Serratia liquefaciens*)				Riedel et al., 2003
		ApeE (*Salmonella* Typhimurium*)*				Carinato et al., 1998
		PlpD (*Pseudomonas aeruginosa*) FplA (*Fusobacteriumnucleatum*)	Vd	Salacha et al., 2010; Da Mata Madeira et al., 2016
						Casasanta et al., 2017

CDI	Growth-inhibition of competing bacteria	CdiA/B (Enterobacteriaceae)			Vb	Diner et al., 2012
		BcpA/B (*Burkholderia pseudomallei*)				Garcia et al., 2013

Alteration of Host		BadA (*Bartonella henselae*)			Vb	Riess et al., 2004
Cell Processes		InvD (*Yersinia pseudotuberculosis*)			Ve	Sadana et al., 2018

Immune evasion	Circumventing host immune response	IgA protease (*Escherichia coli*)			Va	Zinchenko et al., 2018
		EtpA (*Escherichia coli*)			Vb	Roy et al., 2009
		YadA (*Yersinia* spp.)			Vc	Schindler et al., 2012
		Eib (*Escherichia coli*)				Leo and Goldman, 2009; Leo et al., 2011

Cyto-/Hemolysis		VacA (*Helicobacterpylori*)			Va	Cover and Blanke, 2005
		ShlA (*Serratia marcescens*)			Vb	Reboud et al., 2017
		ExlA (*Pseudomonas aeruginosa*)				Yang and Braun, 2000

Adhesin	Adhesion to surfaces/receptors	AIDA-I (*Escherichia coli*)			Va	Laarmann and Schmidt, 2003; Charbonneau et al., 2006
		EhaA (*Escherichia coli*)				
		Pertactin (*Bordetella pertussis*)				Aricò et al., 1993
		FHA (*Bordetella pertussis*)			Vb	Serra et al., 2011
		HMW1/2 (*Haemophilus influenzae*)				Buscher et al., 2006
		YadA (*Yersinia* spp.) Eib (*Escherichia coli*)	Vc	Tertti et al., 1992; Mühlenkamp et al., 2017
						Leo and Goldman, 2009
		Intimin (*Escherichia coli*)			Ve	Kenny et al., 1997
		InvA (*Yersinia* spp.)				Isberg et al., 2000
	
	Auto-Agglutination and biofilm formation	Ag43 (*Escherichia coli*)			Va	Sherlock et al., 2006
		FHA (*Bordetella pertussis*)			Vb	Serra et al., 2011
		EtpA (*Escherichia coli*)				Roy et al., 2009
		YadA (*Yersinia enterocolitica*)			Vc	Trunk et al., 2018
		Eib (*Escherichia coli*)				Leo and Goldman, 2009

Intracellular motility	Activation of actin polymerases	IcsA (*Shigella flexneri*)			Va	Goldberg and Theriot, 1995
		YapV (*Yersinia* spp.)				Besingi et al., 2013
		BimA (*Burkholderia* spp.)			Vc	Benanti et al., 2015

## Topology Of ATs

Autotransporters consist of two distinct regions, a secreted passenger and a β-barrel domain that resides in the bacterial OM. The transmembrane domain typically is C-terminal to the passenger, but in type Ve ATs this domain order is inverted (Figure 1). Both regions are found in a single polypeptide chain with the exception of type Vb secretion systems, where the moieties are separate polypeptide chains (Guérin et al., 2017). While this broad separation into two functional regions is conserved among all type V systems, additional functional features have been identified. Examples include the PL-region (pertactin-like region), stable core or autochaperone region, all describing the same features of the membrane-proximal part of AT passengers that have special functions in folding and transport of the rest of the passenger (Drobnak et al., 2015). To further complicate the issue, the passenger itself has been referred to as the α-domain and the transmembrane β-barrel as the translocator or β-domain (Pohlner et al., 1995; Henderson et al., 2004; Drobnak et al., 2015). To avoid confusion, we will only refer to the β-barrel and the passenger in this review according to Drobnak et al. (2015). In the following section, we will give a short overview over the different structural features of the different sub-classes of ATs.

### Type Va (Classical Autotransporters)

Type Va ATs are commonly known as classical ATs. They have been studied extensively, both functionally and structurally. Well studied members are the IgA protease from *Neisseria meningitidis* and EstA, a lipase from *Pseudomonas aeruginosa* (Henderson et al., 2004). Type Va ATs consist of a 12-stranded β-barrel domain, which functions as a C-terminal anchor in the OM and which is required for the transport of the N-terminal passenger to the extracellular environment. The passenger usually adopts a repetitive β-helix fold extending away from the bacterial cell surface, as demonstrated by the crystal structure of the Pertactin passenger (Emsley et al., 1996). Other forms of passengers are possible as well, as exemplified by EstA folding into a predominantly α-helical passenger (Brzuszkiewicz et al., 2009). The passenger harbors the specific function of the protein, and most model systems that have been studied in different species are important virulence factors. The diversity of passenger functions and specifically of protease functions among type Va passengers has given rise to classifications into SPATE (serine protease autotransporters of Enterobacteriaceae) proteases, SPATE-like and non-SPATE proteases (Yen et al., 2008; Ruiz-Perez and Nataro, 2014). In some cases the passenger domain of type Va ATs can be cleaved off after secretion. Passengers with enzymatic activity, like SPATE proteases, more often belong to the group of cleaved passengers than adhesin passengers, though cleavage has been observed also in adhesins such as AIDA-I (Charbonneau et al., 2006; Barnard et al., 2007; Dautin et al., 2007). Other examples are the SAATs (self-aggregating ATs) such as Ag43 from *E. coli* (Klemm et al., 2004).

### Type Vb (Two-Partner Secretion)

Type Vb secretion systems consist of two distinct polypeptide chains encoded in one operon, e.g., the *Bordetella* filamentous hemagglutinin FHA (Chevalier et al., 2004; Jacob-Dubuisson et al., 2013). Due to this, they are also called two-partner secretion systems (TPSSs). TPSSs are composed of two proteins, one functioning as the translocator (TpsB) and the other as the secreted passenger (TpsA). TpsB is a 16-stranded, OM integral β-barrel protein with two periplasmic POTRA (polypeptide transport-associated) domains (Kajava et al., 2001). Due to the separation of the β-barrel and the passenger into two separate polypeptide chains, the passenger is released into the cell’s environment after transport without any need for release by proteolytic cleavage. The fate of the passenger after secretion can differ. Some TpsB proteins stay attached to the OM in a non-covalently bound manner as exemplified by the *Haemophilus influenzae* proteins HMW1 and HMW2, while others are secreted, such as the *Serratia marcescens* protein ShlA (Braun et al., 1993; St. Geme, 1994).

### Type Vc (Trimeric Autotransporter Adhesins)

Type Vc secretion systems have the same topology as type Va systems, but form highly intertwined trimeric structures. For this reason, and because all examples studied so far are adhesins, they are often referred to as trimeric autotransporter adhesins (TAAs). YadA, the *Yersinia* adhesin A from *Yersinia enterocolitica* and *Yersinia pseudotuberculosis* is the best studied member of this class of ATs (Mühlenkamp et al., 2015). These proteins consist of three identical polypeptide chains, and in their final folded form are composed of a C-terminal 12 stranded β-barrel (4 β-strands per monomer), and a passenger, which is also trimeric and typically folds into a lollipop-like structure with a coiled coil stalk and a globular head domain at the N-terminus of the protein (Hoiczyk, 2000; Linke et al., 2006; Wollmann et al., 2006).

### Type Vd

The type Vd ATs are a fairly recently discovered class of ATs that resembles a hybrid of type Va and type Vb systems with PlpD from *P. aeruginosa* and FplA from *Fusobacterium nucleatum* as prototypical members (Salacha et al., 2010; Casasanta et al., 2017). The C-terminal β-barrel domain of type Vd ATs consist of 16 β-strands which is similar to the β-barrel of TpsB proteins. However, type Vd ATs have only one POTRA domain, whereas TpsB proteins have two POTRA domains for binding their TpsA substrate for secretion (Leo et al., 2012). Whereas type Va ATs have a passenger that typically folds into a β-helical structure, the passenger domains of type Vd ATs, which have been found to harbor lipase activity, adopt an α/β-hydrolase fold (Emsley et al., 1996; Da Mata Madeira et al., 2016). Note though that there are also some type Va ATs with passengers that have an α/β hydrolase fold, e.g., EstA (Brzuszkiewicz et al., 2009). A key difference between Va and type Vd passengers seems to be that type Va passengers have a multitude of different folds and functionalities, while type Vd passengers characterized so far only function as lipases/esterases (Da Mata Madeira et al., 2016; Casasanta et al., 2017).

### Type Ve (Inverse Autotransporters)

Type Ve ATs share obvious similarities to type Va ATs, with a modular architecture including a 12-stranded β-barrel domain and a secreted, monomeric passenger that remains attached after translocation. The major difference to type Va ATs is that the type Ve ATs have an inverted domain order, with the β-barrel at the N-terminal end and the passenger at the C-terminus as shown for intimin and invasin from *Escherichia coli* and *Y. enterocolitica* (Leo et al., 2012, 2015b). This has led to the name “inverse autotransporters” (Oberhettinger et al., 2015). The passenger of inverse ATs typically contains domains with Ig-like or lectin-like folds, and some exemplars have long, repetitive stretches of Ig-like domains that are capped with a lectin-like domain (Leo et al., 2015b). Some type Ve ATs have an additional periplasmic domain which is not found in other types of ATs. This periplasmic domain aids in dimerization as well as in interactions with peptidoglycan, possibly anchoring it and helping in receptor interactions during host invasion (Leo et al., 2015a).

### Type Vf?

The type Vf secretion systems were very recently described as a new class of ATs, with BapA as the prototypical member, and appear to be unique to *Helicobacter pylori*. This proposed class of ATs has a surface-exposed domain inserted into the N-terminal region between the first and second β-strand of an 8-stranded β-barrel domain, and contains no additional passenger at either terminus of the protein. Thus, the proposed passenger actually is an extended loop of the β-barrel domain and the β-barrel is smaller than that of any other AT (Coppens et al., 2018). Though BapA and related proteins have been proposed to be part of the AT family, their topology is very different from the other types of ATs. It is therefore questionable whether these proteins should be considered ATs, and further investigation of the secretion mechanism is required to find out whether these proteins actually self-export in a similar fashion to other ATs.

## Biogenesis of ATs

### Transport Across Membranes and β-Barrel Insertion

Like most OM proteins, ATs follow a conserved pathway in their biogenesis.

Autotransporters are translated in the cytosol where the polypeptide chain is kept in an unfolded state by the help of chaperones and translocated across the inner membrane (IM) into the periplasm by the SecYEG translocon (Sijbrandi et al., 2003; Tsirigotaki et al., 2017). An N-terminal signal sequence ensures proper recognition of the AT as a Sec target, and targeting and secretion through the IM and signal peptide cleavage after transport works in the same way as for other Sec-secreted proteins (Papanikou et al., 2007). Some ATs, like Hbp and AIDA-I, show an extended Sec signal sequence which might aid in slowing down IM translocation and thus in prevention of premature folding and aggregation of the AT within the periplasm (Henderson et al., 1998; Szabady et al., 2005; Jacob-Dubuisson et al., 2013). For type Vb systems, it has been shown that some TpsA passengers aggregate much faster than others and therefore retaining the AT bound to the Sec is beneficial; Otp is a protein which is not prone to aggregation and therefore does not require fast transport to the OM (Choi and Bernstein, 2010). In other systems like FHA, quick secretion is of importance as degradation of unfolded FHA by DegP is more likely due to the length of the FHA precursor (Baud et al., 2009).

In the periplasm, ATs are kept unfolded but in a folding-competent state, shielded from aggregation by periplasmic chaperones like SurA, Skp and DegP (Baud et al., 2009; Ieva and Bernstein, 2009; Oberhettinger et al., 2012; Pavlova et al., 2013; Weirich et al., 2017). Insertion of the β-barrel domain of ATs is then facilitated by the β-barrel assembly machinery (BAM) complex (Jain and Goldberg, 2007; Leo et al., 2012). In *E. coli*, it is composed of five subunits, BamA through BamE. This complex interacts with most if not all OM integral β-barrel proteins (Lee et al., 2018). The 16-stranded β-barrel integral membrane protein BamA helps in insertion of the substrate barrel into the OM by a not yet entirely understood mechanism (Schiffrin et al., 2017). For type Va ATs, it has been clearly shown by crosslinking experiments that the 12-stranded β-barrel membrane anchor folds and inserts into the OM aided directly by the BAM complex. The passenger of EspP, an *E. coli* AT, for example, can be crosslinked to periplasmic chaperones, as well as to its β-barrel domain and to BamA (Ieva and Bernstein, 2009; Pavlova et al., 2013). Similarly, type Vc and Ve ATs interact with the Bam complex, as shown for YadA and Invasin (Roggenkamp et al., 2003; Oberhettinger et al., 2015).

### Passenger Secretion

While most other bacterial secretion systems have access to energy sources like proton gradients across the IM or are directly energized by cytoplasmic ATP, ATs only span the OM, which is too leaky for ion gradients, and the periplasm is devoid of ATP (Nikaido and Vaara, 1985; Silhavy et al., 2006). Various models for how the secretion and folding process of passengers is energized have been proposed. One plausible explanation is that the energy for transport comes from the intrinsic folding capacity of the AT itself, either directly driving export or leading to a Brownian ratchet model where, once secreted, the passenger cannot slide back into the periplasm and is therefore driven to move outside the cell and fold (Henderson et al., 2004; Choi and Bernstein, 2010). Furthermore, asymmetric charge distribution within the passenger has been put forward as a possible driving factor for passenger secretion (Kang’ethe and Bernstein, 2013).

Passenger transport and secretion differ slightly between the various AT subclasses due to differences in domain organization. In type Va ATs, the passenger is transported via a C-terminus-first mechanism. According to the widely accepted hairpin-loop model of secretion, a hairpin-loop is formed at the C-terminus of the passenger in the interior of the β-barrel, followed by sequential folding of the passenger on the cell surface starting from the C-terminus (Junker et al., 2006). This was shown for multiple members of the type Va AT subclass, including Pertactin, Hbp and EspP (Junker et al., 2009; Peterson et al., 2010; Soprova et al., 2010).

For type Vb secretion, models are somewhat different since in the TPSSs the β-barrel domain is separated from the passenger domain. After the TpsB transporter is properly inserted into the OM by the BAM complex, recognition of TpsA by TpsB is provided by interaction of the TpsB POTRAs and the N-terminal TPS signal of TpsA (Baud et al., 2009). The TPS signal is a conserved stretch with an amphipathic character that remains unfolded in the periplasm. Association and dissociation rates of the TPS signal with the TpsB POTRA domains are high based on surface plasmon resonance experiments, making the interaction transient, and helping in later release of the TpsA substrate from its transporter (Delattre et al., 2010; Guérin et al., 2017). NMR experiments have shown similar highly dynamic interactions (Garnett et al., 2015). Crosslinking experiments have further shown that the TPS signal interacts with the TpsB POTRA domains, as well as some central amino acids within the barrel lumen (Baud et al., 2014). Similarly to all other Type V secretion systems, it is assumed that during transport, TpsA is unfolded as it passes through the central pore of the TpsB barrel and that folding of the substrate occurs during exit from the transporter barrel.

There are two different models for how the export of the TpsA is initiated: one is that, like in other ATs, a hairpin is formed within the barrel pore driving folding of the secreted substrate in a C-to-N direction. Release of the TpsB-bound TPS domain would then occur at the end of secretion, after major parts of TpsA have already folded (Pavlova et al., 2013; Norell et al., 2014). In this case, the high on/off rate between the PORTA domains and the TPS signal domain would facilitate the release that is based on the pulling forces generated by the folding process itself (Guérin et al., 2017). According to the second model, the N-terminal TPS domain nucleates folding, i.e., the TPS domain is exported first and the rest of the protein folds N-to-C (Hodak and Jacob-Dubuisson, 2007). The fact that the TpsA proteins’ N-terminal domain can also fold independently bolsters this argument (Clantin et al., 2004, 2007).

In type Vc ATs, passenger secretion is more intricate due to the trimeric nature of the proteins. Three passenger polypeptide chains have to be orchestrated through a comparatively narrow β-barrel domain. After formation of the 12-stranded β-barrel, the passenger is transported to the exterior of the cell starting with the formation of a hairpin loop of each of the three passenger domains followed by folding of the coiled coil stalk (Linke et al., 2006; Szczesny and Lupas, 2008; Mikula et al., 2012; Chauhan et al., 2019). Transport of three distinct polypeptide chains in a hairpin loop conformation across a comparably small barrel might be sterically challenging. The interior of type Vc β-barrels contains many glycine and alanine residues which have small side chains, and it has been suggested that this facilitates passage of multiple chains though the barrel interior (Mikula et al., 2012). Additionally, β-barrel proteins are not necessarily fully rigid pores. The capacity of “breathing” movement without breakage of the hydrogen bonding has already been shown for the usher protein FimD, which in its apo-structure is more narrow than when bound to a transport substrate (Phan et al., 2011). Similar breathing behavior would be necessary in type Vc autotransport to accommodate all chains simultaneously. An additional problem comes with the highly intertwined passenger structure in type Vc systems. Sequential folding after initial hairpin formation would build up mechanical strain. It has been shown for some examples that an YxD/RxD motif toward the C-terminus of the passenger helps in initiation of passenger folding and folding outside the membrane anchor, potentially by releasing mechanical strain. YxD motifs furthermore stabilize right-handed coiled-coils whereas RxD motifs support left-handed coiled-coils (Alvarez et al., 2010). In addition, while the core residues of coiled-coil proteins are generally hydrophobic, some trimeric AT passengers contain hydrophilic residues in these positions. These residues can coordinate anions, which might allow sequences that are otherwise not easily folded to interact and stabilize (Hartmann et al., 2009; Leo et al., 2011).

It is not yet entirely clear how passenger secretion works in type Vd systems, and what role the POTRA domain plays in this (Salacha et al., 2010). It might function either as a chaperone for the passenger, aiding in secretion, or aiding in the recruitment of proteases for passenger cleavage. In some strains of *F. nucleatum* the passenger domain of FplA seems to be cleaved off while in other strains this could not be shown (Casasanta et al., 2017). It is unclear whether proteolytic cleavage of the passenger of type Vd ATs is achieved via autoproteolysis, like in some type Va ATs, or via an independent protease, like in the example of the NalP cleaving the type Va AT IgA protease for release from its β-barrel domain (Salacha et al., 2010; Casasanta et al., 2017). However, the fact that type Vd passengers remain uncleaved in some strains and when heterologously expressed in *E. coli* supports the latter interpretation (Salacha et al., 2010).

The biogenesis of type Ve ATs is similar to the one of type Va ATs. Although the topology of type Ve ATs is inverted, the β-barrel functions as a transport pore in an analogous way via formation of a hairpin-loop, and the passenger is secreted in a very similar fashion to the passenger secretion of classical ATs, but in the opposite direction (N-to-C rather than C-to-N) (Oberhettinger et al., 2012, 2015). Folding is energized by sequential folding of the extracellular Ig-like domains, as shown for the example of Intimin (Leo et al., 2016).

## Functions of at Passengers

**FIGURE 2:**
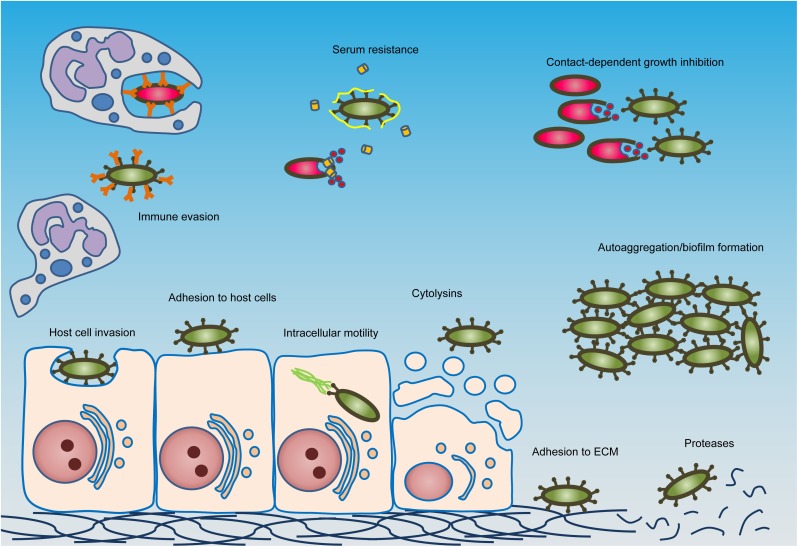
Typical activities of autotransporter proteins. The major activities found in autotransporters are shown schematically. Green bacteria express autotransporters, but red bacteria do not. Autotransporters mediate activities such as adhesion to host cells or the extracellular matrix (ECM), invasion of host cells, immune evasion and serum resistance, contact-dependent growth inhibition, toxicity toward host cells, intracellular mobility, protease activity, and autoaggregation and biofilm formation. Cytolysin activity can be mediated by lipases or pore-forming toxins. Immune evasion can be accomplished by a variety of mechanisms, such as by binding to the constant regions of antibodies to prevent opsonisation (depicted here), cleavage of antibodies, mediating phagocytosis resistance or modulating immune cell signaling and gene regulation. In serum resistance, bacteria become resistant to the bactericidal effects of complement (shown as orange cylinders) by binding to complement-regulatory factors such as Factor H (shown as yellow ribbons). For full descriptions, see main text.

Research on ATs has traditionally focused on a single protein and its function (often in pathogenesis) (Figure 2), or on individual subclasses based on topology and biogenesis (Figure 1). The latter has led to the systematic sub-classification into type Va to Ve secretion systems, but this classification does not reflect the function of the passenger. Passengers from different subclasses can have similar functions despite structural differences, and some individual passengers mediate multiple functions. Therefore, a systematic differentiation between passenger functions is harder to achieve (Dautin and Bernstein, 2007; Drobnak et al., 2015). Furthermore, while some functions can be described as general virulence traits and can be found in a wide variety of Gram-negative bacteria, such as protease activity (Yen et al., 2008; Dautin, 2010), other functions are rather unique and are involved in tasks specific to the bacterium and its lifestyle, such as intracellular mobility and nutrient acquisition in limited environments (Luckett et al., 2012; Benanti et al., 2015).

Tables 1, 2 show an overview of the functions and exemplary members of the functional groups.

**Table 2:** Adhesins and adhesion targets of autotransporters.

Type	Host bacterium	Binding partners	References
**Va**			
AIDA-I	*Escherichia coli*	glycoprotein	Laarmann and Schmidt, 2003
EhaB		collagen, laminin	Chagnot et al., 2012
Tsh		collagen, fibronectin	Kostakioti and Stathopoulos, 2004
Hap	*Haemophilus influenzae*	fibronectin, laminin, collagen	Fink et al., 2003
MisL	*Salmonella enterica*	collagen, fibronectin	Dorsey et al., 2005
ShdA			Kingsley et al., 2002, 2004
**Vb**			
FHA	*Bordetella* spp.	heparin, dextran	Menozzi et al., 1994
EtpA	*Escherichia coli*	flagellin	Roy et al., 2009
HMW1/2	*Haemophilus influenzae*	sialylated glycoprotein/ unknown	St. Geme, 1994; St. Geme and Yeo, 2009
MhaB1	*Moraxella catarrhalis*	mammalian cells	Balder et al., 2007
HrpA	*Neisseria meningitidis*	mammalian cells	Schmitt et al., 2007
**Vc**			
BadA	*Bartonella henselae*	collagen, laminin, fibronectin	Riess et al., 2004
EhaG	*Escherichia coli*	laminin, fibronectin, fibrinogen, collagen	Totsika et al., 2012
UpaG			Valle et al., 2008; Totsika et al., 2012
DsrA	*Haemophilus ducreyi*	fibronectin	Leduc et al., 2008
NcaA		Collagen	Fulcher et al., 2006
Hia	*Haemophilus influenzae*	cells	Spahich, 2011
UspA	*Moraxella catharralis*	fibronectin, laminin	Tan et al., 2005, 2006
NadA	*Neisseria meningiditis*	cells	Tavano et al., 2011
NhhA		laminin, heparan sulfate, cells	Scarselli et al., 2006
SadA	*Salmonella typhimurium*	cells (no ECM)	Raghunathan et al., 2011
YadA	*Yersinia enterocolitica*	collagen, fibronectin, laminin	El Tahir and Skurnik, 2001
**Ve**			
FdeC	*Escherichia coli*	cells, collagen	Nesta et al., 2012
Intimin		Tir protein	Batchelor et al., 2000
Invasin	*Yersinia* spp.	β1-integrins	Isberg et al., 2000
InvD		IgG/IgA	Sadana et al., 2018

### Enzymatic Activities

Passengers with enzymatic functions can directly alter host cell processes, be involved in immune evasion or help in the establishment and colonization of niches. The large diversity of possible enzymatic functions includes protease activity, lipase activity, but also contact-dependent growth inhibition (CDI) mediated by ADP ribosyl cyclases, adenosine deaminases, or nicking endonucleases.

#### Lipases and Esterases

Lipase and esterase activity can be found in type Va as well as in type Vd passenger domains. It has been proposed that these enzymes aid in niche establishment especially for intracellular bacteria, but also in alteration of host cell signaling by phosphoinositide (PI) cleavage (Casasanta et al., 2017). Though there are some ideas and models for the role that ATs with lipase and esterase function play in virulence, their exact function is not clear, and it is quite possible that AT lipases of different bacteria act on different targets in the host (Da Mata Madeira et al., 2016; Casasanta et al., 2017).

A prototypical example for a passenger with a lipase domain is EstA, a type Va AT of *P. aeruginosa.* In this protein, the passenger is not cleaved from its β-barrel domain (Wilhelm et al., 1999). EstA is implicated in the cleavage of rhamnolipids, which by themselves are important for biofilm formation and have toxic properties (Davey et al., 2003; Klausen et al., 2003). When deleting the *estA* gene, rhamnolipids in biofilms shifted from di-rhamnolipids to mono-rhamnolipids, indicating that rhamnolipids might be a target of EstA (Wilhelm et al., 2007; Tielen et al., 2010). Knockouts of *estA* also showed alterations in motility. While swimming and swarming are absent in *estA* deletion strains, twitching motility is enhanced, which seems somewhat puzzling taking into account that only swarming, not swimming or twitching motility is influenced by the rhamnolipid content of a biofilm (Kohler et al., 2000; Déziel et al., 2003; Wilhelm et al., 2007). Other differences in motility can thus not be explained by rhamnolipids as targets of EstA alone (Wilhelm et al., 2007). Other type Va AT with comparable lipolytic functions can be found in *Moraxella catarrhalis* (Mcap), *Serratia liquefaciens* (EstA) and also *Salmonella typhimurium* (ApeE) (Carinato et al., 1998). EstA of *S. liquefaciens*, for example, is involved in cellular signaling by providing the cells with enough lipids for synthesis of second messenger molecules (Riedel et al., 2003). This function could not be verified for EstA in *P. aeruginosa*, however (Wilhelm et al., 2007).

In type Vd ATs, all passengers described so far have lipase activity. Though lipase activity can also be found in type Va ATs, the structure and domain organization of type Vd ATs is very distinct with a single POTRA domain fusing the β-barrel and the passenger even though both passengers have a α/β hydrolase fold (Salacha et al., 2010; Casasanta et al., 2017). Furthermore, the catalytically active site in type Va lipases like the GDSL lipase EstA usually consists of a catalytic triad while type Vd ATs show a catalytic dyad (Desvaux et al., 2005; Brzuszkiewicz et al., 2009; van den Berg, 2010). Prototypic for type Vd ATs are PlpD from *P. aeruginosa* and FplA from *F. nucleatum* (Salacha et al., 2010; Casasanta et al., 2017). The name of the *Pseudomonas* proteins comes from structural similarity of the passenger to patatin from potatoes; hence the name patatin-like protein D. So far more than 200 Gram-negative bacterial species have been found to encode PlpD orthologues, including a wide variety of pathogenic as well as non-pathogenic, environmental bacteria (Salacha et al., 2010). The patatin-like domain of PlpD harbors A1 phospholipase activity (Da Mata Madeira et al., 2016). Similarly, the PlpD homologue from *F. nucleatum*, FplA, has phospholipase A1 activity and displays very high hydrolytic activity toward artificial substrates (Casasanta et al., 2017). The structure of the PlpD passenger has been solved, and the active site offers enough space to accommodate C16 to C20 acyl chains (Da Mata Madeira et al., 2016). The catalytic dyad in PlpD consists of Ser60 and Asp207, which are in close proximity to a hydrophobic helix within the cleft containing the active site, which probably stabilizes the lipid interaction (Casasanta et al., 2017). Similarly, in FplA, the catalytic residues are Ser98 and Asp243 (Casasanta et al., 2017). Biochemical experiments with FplA showed no binding affinity for phospholipids like phosphatidycholine (PC), phosphatidyethanolamine (PE), and phospatidic acid (PA) but it seems to bind to phosphatidyinositol (PI) phosphates which acts in signaling of host cells (Casasanta et al., 2017). It has been proposed that lipase activity is important for establishing a niche especially for intracellularly living bacteria by recognizing phosphorylated PIs and by their cleavage altering cell signaling (Casasanta et al., 2017). Furthermore, this lipase activity might also help in cytosolic release of the bacteria from the vacuole and phagosomal survival within the host cell, which are important for niche establishment and an intracellular lifestyle of this bacterium (Casasanta et al., 2017).

#### Proteolytic Activity

Proteolytic cleavage of host target proteins can be beneficial for the bacterium in terms of virulence. A number of proteolytically active type Va AT passengers have been discovered. The first AT discovered is Immunoglobulin A (IgA) protease from *Neisseria gonorrhoeae* and *N. meningitidis.* The passenger of IgA protease can be divided into a 106 kDa protease domain, a more C-terminal 3 kDa γ-peptide, a 12–44 kDa α-peptide, a linker region and the C-terminal β-barrel membrane anchor (Halter et al., 1984). Following export, the passenger is cleaved off from the β-barrel transporter to function as a protease in virulence. Cleavage of the passenger can happen in different ways: either the passenger is autoproteolyzed or cleaved by another AT called NalP in a phase-dependent fashion (Roussel-Jazédé et al., 2010). NalP-dependent cleavage results in the release of the full passenger domain, including the protease domain, the α-peptide, the γ-peptide and the linker region, while autoproteolysis results in the release of fragments of different sizes: either the protease, the protease and the γ-peptide or the complete passenger. The autoproteolytic cleavage seems to be strain-dependent and can have different effects on the role the released passenger has in virulence.

More recently, it has been shown that IgA protease can also modulate host gene expression (Hulks and Plaut, 1978). If the passenger is cleaved off together with the α-peptide, the α-peptide via its nuclear localization-like sequence can guide the IgA protease domain to the host cell nucleus. The IgA protease domain can then cleave NF-κB and p65/RelA in the nucleus and thereby modulate the entire host cell response to pathogenic stressors (Pohlner et al., 1995; Besbes et al., 2015).

Furthermore, IgA proteases have been shown to cleave LAMP1, a highly glycosylated endosomal and lysosomal membrane protein which normally protects the membrane from degradation (Hauck and Meyer, 1997). Degradation of LAMP1 in turn seems beneficial for the growth of intracellular bacteria, as *Neisseria* lacking IgA protease grow slower intracellularly than wild-type bacteria (Lin et al., 1997). *Neisseria* pilus proteins and porins influence the calcium intake of epithelial cells, leading to the exocytosis of endosomes (Ayala et al., 2002). This shows that IgA proteases secreted by *Neisseria* have multiple targets and play an important role in survival and growth of *Neisseria* in infection contexts.

Autotransporter proteases do not only play a role in host immune response circumvention and establishing a niche in the host, but also in nutrient acquisition. As an example, AaaA from *P. aeruginosa* is involved in nitrogen acquisition from peptides in chronic infections. It acts as an aminopeptidase aiding in long-term infections in mice (Luckett et al., 2012). Another group of type Va ATs with protease activity are the SPATEs (Serine Protease Autotransporters of *Enterobacteriaceae*) proteins. Their targets can be very diverse; one prominent example is Hbp (hemoglobin protease or hemoglobin binding protein) from *E. coli.* Hbp employs an active Ser residue for cleavage of hemoglobin, but shows no specificity for other proteins such as albumin (Otto et al., 2005). Hbp, like all SPATEs, is cleaved off after passenger transport (Dautin, 2010). Functionally, Hbp is used by enterohemorrhagic *E. coli* strains for heme acquisition (Dautin, 2010).

### Contact-Dependent Growth Inhibition (CDI)

Enzymatically active passenger domains can also be found in type Vb ATs. Like in the type Va ATs, there can be dramatic differences in enzymatic functions. One major function of the passengers of type Vb ATs is CDI. Here, the passenger domains can function as nucleases, deaminases, and also as metallopeptidases. The name of this functional class of type Vb ATs is potentially misleading, since it is still unclear whether growth inhibition of competing bacterial strains is the main evolutionary purpose of this system (Guérin et al., 2017).

Contact-dependent growth inhibitions are transporter-effector pairs called CdiB/A pairs, where CdiA corresponds to TpsA and CdiB to TpsB (see section on type Vb transport, above) (Ruhe et al., 2013a). Upon secretion of CdiA by CdiB, CdiA acts as a toxin that can inhibit the growth of other bacteria, typically of the same species. In order to render themselves immune against their own CdiA toxin, the *cdi* gene cluster encodes an additional immunity protein named CdiI (Aoki et al., 2005). The receptor for *E. coli* CdiA is BamA, the β-barrel protein of the BAM (Aoki et al., 2008). BamA is an essential protein in all bacterial species, but shows considerable sequence variability in its extracellular loops, allowing discrimination between closely and more distantly or unrelated species for CDI (Ruhe et al., 2013b).

Growth inhibition by the cytotoxic C-terminus of CdiA (Cdi-CT) is then facilitated by diverse mechanisms such as nuclease activity, adenosine deaminase activity, metallopeptidase activity, or ADP ribosyl cyclase activity (Ruhe et al., 2013a). One well-studied example of a CDI system is the system of *E. coli* UPEC 536, which encodes a CdiA-CT that harbors tRNase activity (Aoki et al., 2010). Interestingly, this protein is not active until in its host, where it binds to CysK, a protein involved in the catalysis of L-serine to L-cysteine (Diner et al., 2012). Only upon binding to CysK is CdiA able to cleave tRNA. Normally, CysK interacts with CysE, but CdiA has a common amino acid motif with CysE that allows it to bind CysK and become active (Diner et al., 2012).

Another example of a CDI system is the *cdiAIB* system of *Burkholderia pseudomallei* coding for BcpAIB. Here, BcpA is the secreted protein that plays a role in cooperative bacterial communication during biofilm formation. Architecturally, BcpA also harbors a toxic C-terminus, but secretion of BcpA enhances the formation of stable biofilms, possibly by influencing the amount of extracellular DNA as part of the stable biofilm. It has also been proposed that BcpA has nickase activity which might consolidate the microbial community in a biofilm by crosslinking eDNA or attaching the bacteria to eDNA within a biofilm (Garcia et al., 2013; Ruhe et al., 2013a). This example suggests that CDI systems not only provide a growth advantage over other bacterial strains in a competitive environment, but can also play more complex roles.

### Immune Evasion

Immune evasion is a collective term for a number of highly specialized mechanisms employed by some bacteria to escape from host immune responses. ATs can participate in immune evasion by interfering with different components of the host immune system.

The first level of immune evasion is serum resistance, which means the ability to survive the action of the complement system that is part of the innate immune response. Serum resistance is achieved by binding to and/or inactivating different components of the complement cascade, such as by binding to Factor H and C3 through C9 products. Binding complement proteins via adhesins like the YadA from *Y. enterocolitica* or the classical AT Vag8 from *Bordetella pertussis* inhibit the full cascade and thus formation of the terminal complement complex which would lead to lysis of the pathogen (Marr et al., 2011; Schindler et al., 2012). YadA is able to sequester different factors, like Factor H and C4b-binding protein, involved in complement regulation (Grosskinsky et al., 2007; Biedzka-Sarek et al., 2008; Kirjavainen et al., 2008). In addition, YadA can bind directly to C3b and iC3b, which in turn promotes Factor H binding and allows *Y. enterocolitica* to escape killing by complement (Schindler et al., 2012). YadA-expressing *Yersinia* can also bind to a variety of host surfaces, initiating virulence processes like secretion of *Yersinia* outer proteins (Yops) via the type III secretion system and thereby killing host immune cells like neutrophils (Rosqvist et al., 1991; Bliska et al., 1993).

Another, more indirect means of host immune evasion is the disguise of immunogenic structures on the bacterial surface. EtpA from *E. coli* for example binds to flagella, which may serve in protection against the host immune system by covering FliC, the main flagellar protein, as an antigen; this may help the bacterium to colonize the host (Roy et al., 2009). EtpA serves a double purpose here, as it also mediates binding to host surfaces and thus bacterial adhesion and biofilm formation (Roy et al., 2009). ATs can also bind factors of the adaptive immune response. The Eib proteins from *E. coli* evade the host immune system by binding to Fc antibody fragments of IgG, possibly in order to avoid opsonization and subsequent phagocytosis (Leo and Goldman, 2009). A more prominent and well-studied example for interactions of ATs with the adaptive immune system is IgA protease of *Neisseria* spp. Here, the serine protease domain exerts endopeptidase activity, recognizing and cleaving the TPPTPSPS motif in the hinge region of human IgA1 and IgA2 and releasing the antigen-binding Fab region from the Fc region. This allows the bacteria to evade opsonization (Plaut et al., 1975, 1977).

### Alteration of Other Host Cell Processes

Autotransporters can also influence other host processes beyond the host immune response, typically to promote pathogenic processes. A well-studied example is the TAA BadA, which activates hypoxia-inducible factor 1 (HIF-1), which in turn leads to the release of vaso-endothelial growth factor (VEGF) inducing endothelial proliferation (Kempf et al., 2001). Interaction of BadA-expressing *Bartonella henselae* with endothelial cells has been shown to inhibit apoptosis, supporting the effect of VEGF induced vaso-endothelial proliferation (Kempf et al., 2005). Interestingly, a comparative study investigating the interplay of BadA with the type IV secretion system VirB/D4 in different clinical isolates showed that the enormous length of the BadA stalk (240 nm) might interfere with injection of toxic proteins into the host cell by the type IV secretion system in *B. henselae* (Riess et al., 2004; Müller et al., 2011). This at first seems puzzling, but in light of the intracellular lifestyle of these bacteria, encouraging endothelial growth and inhibiting apoptosis as a natural host cell reaction to infection would benefit the bacteria in a way that the bacteria promote host cell growth for intracellular replication (Schülein et al., 2001).

Alterations in host immune responses not only benefit bacteria with an intracellular lifestyle but also bacteria invading deeper tissues, such as some *Yersiniae*. InvD from *Y. pseudotuberculosis* has recently been shown to utilize host antibody binding during acute infections of the intestinal tract for virulence (Sadana et al., 2018). InvD is a type Ve AT which specifically binds to the Fab region of IgA antibodies. This binding might enable InvD expressing *Yersinia* to alter the host immune response. This way immune exclusion, normally preventing bacteria from crossing the mucosal barrier, can be circumvented and thus allows the bacteria to invade deeper tissues (Sadana et al., 2018). Similarly, the *Shigella flexneri* AT SIgA is involved in invasion of the Peyer’s patches, though *Shigella* is not usually found in underlying tissues (Mantis et al., 2002; Favre et al., 2005).

### Cyto- and Hemolysins

Passenger domains functioning as cyto- and hemolysins are so far mostly known in type Vb ATs. Here, toxins like ShlA from *S. marcescens* and ExlA from *P. aeruginosa* are secreted and lead to leakage from or lysis of host cells. The cyto- and hemolytic activity of the TpsA C-termini is most often conferred by pore formation within the target cell membrane, triggering a cascade of downstream events that cause the cells to lose their intercellular integrity and to die of ATP depletion. Both ShlA and ExlA form pores in epithelial and endothelial cells causing a massive influx of cations that reduces or abolishes the membrane potential. This leads to ATP depletion and to the activation of eukaryotic proteases cleaving cadherin, leading to breakage of the epithelial barrier (Hertle et al., 1999; Reboud et al., 2017). A classical AT with a cytotoxic passenger can be found in *H. pylori* and is named VacA (vacuolating cytotoxin A). The passenger of VacA is cleaved off and acts in induction of host cell vacuolation, which does not seem to be lethal for the host cell but is important for efficient colonization of the bacteria within the host (Cover and Blanke, 2005). VacA can also act on mitochondria and thus induce apoptosis as part of VacA cell toxicity (Cover and Blanke, 2005).

### Adhesion

Adhesion to host cells is a major attribute of many different ATs. Interactions with host cells are usually conferred by adhesins belonging to ATs of types Va, b, c, and e. Importantly, “adhesin” serves as a collective term for different types of interactions, with host cells, tissues, or non-living surfaces. In pathogenesis, depending on the individual adhesin and host organism, this can range from diffuse adhesion to a variety of surface molecules to very specific, high-affinity interactions with a given receptor as shown in Table 2.

#### Adhesion to Surfaces

In order for bacteria to thrive in a competitive environment or during an ongoing infection, the ability to adhere to biotic and abiotic surfaces is pivotal to the bacteria, both in direct contact with host cells and as a first step toward biofilm formation. Being able to adhere to a surface ensures not only interaction with a target cell or surface but also helps the bacteria to regulate gene expression optimal for biofilm conditions. AIDA-I is a major adhesin in *E. coli* that belongs to the class of classical ATs (Benz and Schmidt, 1989). It has been shown to confer diffuse adhesion to human cells and also to other cell types by interaction with surface glycoproteins of the host cells (Laarmann and Schmidt, 2003; Sherlock et al., 2004). Both the receptors and AIDA-I itself are glycosylated. The protein responsible for glycosylation of AIDA-I is encoded directly upstream of the *aidA* gene and is named *aah* (Benz and Schmidt, 2001). Without glycosylation, AIDA-I does not function as an adhesin anymore, though glycosylation appears to be dispensable for autoaggregation, another virulence trait conferred by AIDA-I (Benz and Schmidt, 2001; Sherlock et al., 2004). The passenger of AIDA-I can be cleaved *in vitro*, and it has been hypothesized that cleavage of the passenger might contribute to persistence of the bacterial infection as released AIDA-I passengers facilitate host cell entry (Charbonneau et al., 2006; Pizarro-Cerdá and Cossart, 2006).

In contrast to the diversity of target structures of AIDA-I, the type Vb adhesins FHA from *B. pertussis* and EtpA from *E. coli* target a smaller set of host cell structures in order to confer adhesion. FHA binds specifically to certain surface glycans in human lung epithelial cells (Tuomanen et al., 1988; Relman et al., 1990). FHA favors some carbohydrates over others on the surface of ciliated cells, leading to adhesion to the target and later infection of the lung epithelial cells. Also, EtpA has a more specific mode of action by binding to flagellin, as described in Section “Immune Evasion” (Roy et al., 2009).

Adhesin functions have also been found in essentially all type Vc ATs studied so far, leading to the term trimeric autotransporter adhesin (“TAA”) (Linke et al., 2006). The best-studied adhesin belonging to this group is YadA, the *Yersinia* Adhesin A. This protein is encoded on the 70 kb pYV virulence plasmid in all human pathogenic *Yersinia* species including *Y. enterocolitica*, *Y. pseudotuberculosis*, and *Y. pestis*. However, in *Y. pestis* YadA is not expressed at all due to a frameshift in its gene (Rosqvist et al., 1988; Skurnik and Wolf-Watz, 1989), and the role of *Y. pseudotuberculosis* YadA has been elusive since it seems to be dispensable for full virulence (Bolin and Wolf-Watz, 1984). In *Y. enterocolitica*, YadA is important for a multitude of virulence-associated traits. The YadA β-roll head domain at the very N-terminus of the passenger plays an important role in binding to different ECM proteins in the host (El Tahir and Skurnik, 2001). YadA associates with general binding to ECM molecules, including collagen, laminin, fibronectin, and vitronectin (Emody et al., 1989; Flügel et al., 1994; El Tahir et al., 2000; Heise and Dersch, 2006; Mühlenkamp et al., 2017). The interaction of YadA with collagen relies on binding to a triple-helical structure of collagen rich in iminoacids and poor in charged residues (Leo et al., 2008). Because of the high density of YadA on the cell surface during infections it is possible for YadA to bind to collagen strongly despite the low affinity of the interaction (Leo et al., 2010).This contributes to host cell attachment and tissue invasion. For the latter, expression of both YadA and Invasin seems important (Bliska et al., 1993; Eitel et al., 2002). Entry into epithelial cells is needed to further disseminate to underlying tissues and to Peyer’s patches and subsequent infection of the liver and spleen (Isberg et al., 1987). BadA from *B. henselae* is among the largest characterized trimeric AT adhesins, with an overall length of 240 nm and a molecular weight of 984 kDa of the trimer (Riess et al., 2004). Similarly, to YadA, BadA binds to ECM proteins including collagen, fibronectin, and laminin (Goldman and Linke, 2011; Müller et al., 2011). The stalk domain of BadA is important for binding to fibronectin, as BadA mutants lacking most of the stalk cannot bind fibronectin anymore (Kaiser et al., 2012). Apart from the stalk binding to fibronectin, most of the adhesive properties lie within the BadA head domain (Müller et al., 2011; Kaiser et al., 2012).

#### Adhesion to Receptors

Not all ATs bind to multiple surfaces in a promiscuous fashion. Some ATs also bind to specific host receptors, or even to bacterial receptors injected into the host. Intimate contact initiated by Intimin, an *E. coli* type Ve AT, employs such a bacterial receptor inserted into the host membrane named Translocated intimin receptor (Tir). During infection of the small intestine of the host, enteropathogenic *E. coli* and enterohemorrhagic *E. coli* (EHEC and EPEC) induce attaching and effacing (A/E) lesions of the mucosal membrane. These A/E lesions are characterized by polymerization of actin and other cytoskeletal proteins leading to the demise of the cells and at the same time to building an actin pedestal for adhesion of the pathogen. In order for the bacterium to achieve this infection platform, EHEC and EPEC genomes include a pathogenicity island termed LEE (locus of enterocyte effacement), which among other factors encodes for the Tir receptor and intimin (Kenny et al., 1997; Deibel et al., 1998; Gauthier et al., 2000). Though the exact mechanism by which the Tir receptor is inserted into the host cell plasma membrane is still unclear, host cell delivery is facilitated by the bacterial type III secretion system, which is also encoded within the LEE island (Gauthier et al., 2000; Frankel et al., 2001). While Tir is important as a receptor for the Intimin passenger, the Intimin-Tir complex also has implications in cytoskeletal dynamics in the host cell and is thus a multifactorial virulence factor of *E. coli* (Kenny, 1999; Goosney et al., 2001).

Another important member of the type Ve ATs is Invasin of *Yersinia* spp. In contrast to Intimin, it does not bind via a bacterial receptor in the host cell, but instead binds directly to β_1_-integrins expressed on the apical side of gut epithelial cells (Isberg et al., 1987, 2000; Schulte et al., 2000). As a consequence of this binding, *Yersinia* spp. are internalized into the cell via endocytosis and can infect the underlying tissue (Isberg and Leong, 1990; Pepe, 1993; Grassl et al., 2003). Subsequently, Invasin-expressing bacteria can infect the lymph nodes and disseminate into other tissue types (Isberg et al., 2000).

Other subclasses of ATs can also use specific receptors expressed on host cells for adhesion. UspA1, a *M. catarrhalis* TAA, binds to carcinoembryonic antigen-related cell adhesion molecule 1 (CAECAM-1), a cell surface protein displayed by epithelial cells (Hill and Virji, 2003). Interestingly, UspA1 is an example of an AT where the binding function lies within the coiled-coil stalk of the protein (Conners et al., 2008). Due to the length of UspA1 and the density with which UspA1 covers the surface of *M. catarrhalis*, this interaction requires bending of the stalk in order to interact with CAECAM-1 as a receptor (Conners et al., 2008). In addition to the interaction with CAECAM-1, UspA1 also binds to laminin and fibronectin via the head domain in a similar fashion as other type Vc ATs (Tan et al., 2005).

#### Autoaggregation and Biofilm Formation

Biofilms act as a protective mesh that protects bacterial communities from outside influences. Biofilms are a lifestyle that is distinct from that of planktonic bacteria; this is reflected in very different gene expression patterns and by signaling processes inside the biofilm that lead to this adaption. Once established, biofilms can be very hard to remove and are a special challenge in infection and hygiene contexts (Monds and O’Toole, 2009; Trunk et al., 2018).

Both initial adhesion to surfaces and autoaggregation are necessary for establishing biofilms on surfaces. A model system for self-recognition leading to autoaggregation of the bacteria is Ag43 (Antigen 43), a type Va AT from *E. coli*. Ag43 confers intercellular binding by a self-recognizing handshake interaction. By this mechanism the bacteria flocculate, which is beneficial in colonization, immune evasion and persistence in the host (Klemm et al., 2004; Sherlock et al., 2006).

Also FHA aids in the formation of biofilms and thus directly contributes to host colonization and persistence during pathogenesis in *B. pertussis*, together with other proteinaceous factors regulated by the BvgAS system, like fimbriae and ACY, which negatively regulates biofilm formation by interaction with FHA (Irie et al., 2004). In this context, FHA seems to be important in initiation of the attachment of bacteria to the surface, thereby aiding in building micro-colonies that later become part of a bacterial biofilm. FHA also seems to play a major role in maintaining the integrity of the biofilm (Serra et al., 2011). Surprisingly, free FHA actually inhibits the formation of biofilms. This has been speculated to play a regulatory role in biofilm formation during pathogenesis of *B. pertussis* (Serra et al., 2011). In *E. coli*, the type Vb protein EtpA plays a major role in adhesion and biofilm formation. In contrast to free FHA that regulates biofilm formation, secreted EtpA plays a bridging role, aiding in the interaction of flagella with intestinal cells during enterobacterial infection. EtpA binds conserved domains of the flagellar protein FliC at the tip of the flagellum. EtpA then guides the bacterium to gut epithelial cells, where it interacts with mucin producing cells and aids in biofilm formation (Roy et al., 2009).

YadA form entero-pathogenic *Yersiniae* contributes to the induction of microabscesses by autoaggregation. Bacteria expressing YadA tend to aggregate following a zipper-like interaction of YadA proteins displayed on the bacterial surface. This autoaggregation seems beneficial during infection of the Peyer’s patches since induction of microabscesses aids in bacterial persistence (El Tahir and Skurnik, 2001). Autoaggregation by YadA also seems to play a role in general biofilm formation, which in turn contributes to persistence of the infection and immune evasion (Trunk et al., 2018). Thus, YadA is a premier example of how trimeric AT adhesins can take part in multiple virulence-associated tasks including adhesion, immune evasion, autoaggregation, and biofilm formation.

#### Adhesion to Abiotic Surfaces

Adhesion to abiotic surfaces such as rubber, glass, or plastic is a major problem when it comes to working with primary, sterile material in hospital settings or the food industry as adhesion to surfaces is the first step in formation of stable biofilms (Lindsay and von Holy, 2006). Some ATs are universally sticky and interact with a variety of surfaces. Especially TAAs, for example YadA and BadA, interact with various surfaces such as plastic and glass (Schulze-Koops et al., 1993; Müller et al., 2011; Berne et al., 2015). This depends on various factors including the nature of the surface as well as the growth conditions (static vs. flow). There are also specific differences: BadA, for example, interacts more strongly with plastic than YadA (Müller et al., 2011). Together with other modes of surface adhesion and auto-aggregation, YadA, and BadA can thus induce formation of biofilms on a variety of materials. Another well-studied TAA, AtaA from *Acinetobacter* sp., does not appear to have specific binding partners. Like YadA, AtaA has been shown to function in adhesion to collagen and laminin but in general displays low specificity for any particular substrate – instead, it seems to be “universally sticky” adhering strongly to a variety of biotic and abiotic surfaces, including, e.g., polyurethane, steel, and glass (Ishikawa et al., 2012; Hori et al., 2015; Koiwai et al., 2016). Similar features are known for YeeJ, a type Ve inverse AT from *E. coli* involved in adhesion to numerous abiotic surfaces and biofilm formation (Moriel et al., 2017). Unfortunately, though many AT adhesins exhibit the ability to interact with abiotic surfaces, information regarding affinities and systematic studies on which surface materials are preferred are sparse.

## Intracellular Motility

Some bacteria have the ability to invade cells and live within the host cells. This is beneficial for virulence because the bacterium is protected from host immune responses, has access to nutrients, and is influenced less by the harsh environment within the host. Examples of ATs involved in this process have already been mentioned above, including YadA and IgA protease that are implicated in tissue invasion and in release into the host cytosol after uptake into the host cells, respectively. Once inside the cell, bacteria can use ATs in different ways to confer intracellular motility employing the host cell actin cytoskeleton. This motility can be conveyed through the interaction with actin polymerases of the host or by mimicking polymerizing factors themselves (Sitthidet et al., 2011). Both mechanisms can be found in BimA, a type Vc AT of *B. pseudomallei*, *Burkholderia mallei*, and *Burkholderia thailandensis* (Stevens et al., 2005). The mechanism employed for actin polymerization differs slightly between the different species. *B. mallei* and *B. pseudomallei* BimA have a WH2 domain (Wiskott-Aldrich syndrome protein homology domain) that directly mimics WASP (Wiskott-Aldrich syndrome protein) as an actin polymerase. The *B. thailandensis* BimA has a CA domain (central and acidic) that can bind and activate Arp2/3, a complex that activates actin polymerization, in addition to a WH2 domain (Sitthidet et al., 2011; Benanti et al., 2015).

A slightly different mechanism for actin polymerization is used by IcsA, also called VirG, a type Va AT of *S. flexneri* and YapV, a type Va AT of *Y. pseudotuberculosis* and *Y. pestis*. While BimA either binds Arp2/3 by mimicking WASP through a WH2 domain or activate actin polymerization directly by a CA domain, IcsA and YapV seem to interact with N-WASP (neural WASP) (Besingi et al., 2013; Chauhan et al., 2016). IcsA is mostly located at the bacterial pole (Goldberg et al., 1993). Its passenger binds to N-WASP which then can activate Arp2/3 which subsequently functions as an actin polymerase (Bernardini et al., 1989; Goldberg and Theriot, 1995). YapV from *Y. pestis* functions in a similar fashion. Like IcsA, YapV also binds to N-WASP, an actin polymerization factor in order to influence actin polymerization and utilize it for intracellular movement (Besingi et al., 2013; Chauhan et al., 2016; Leupold et al., 2017).

## Conclusion and Outlook

The different subclasses of type V secretion systems, or ATs, display similarities in their biogenesis and mode of passenger secretion, but the functional implications of their passengers in virulence as well as in symbiosis are very diverse. These functions can extend from surface adhesion via enzymatic activity to complex interactions with cellular factors directly influencing host cell behavior. These diverse functions do not cluster with the secretion system (sub)classification; similar functions can be found across some or all subclasses. Detailed mechanistic knowledge about functions of AT passengers is available only for a few well-studied examples, and while the biogenesis pathway(s) are conserved across all species that harbor AT genes, the specific AT functions are often not.

Though well studied, open questions remain in the biogenesis of ATs. It is not entirely clear how the β-barrel domain is inserted into the membrane, and specifically, what role the BAM complex plays in this. BAM is involved in the insertion of all β-barrel membrane proteins in the OM of Gram-negative bacteria, but several AT biogenesis models suggest additional functions for BAM also in passenger secretion (Sauri et al., 2009; Leo et al., 2012; Leo and Linke, 2018). Partly as a consequence of this debate, the mechanism of passenger secretion by the β-barrel is not fully understood. While type Va and Ve ATs secrete their passengers via a hairpin-loop intermediate, the question of how this works, e.g., in trimeric ATs is still under debate (Leo and Linke, 2018). As to whether all three passenger polypeptides are transported at the same time or sequentially has still to be shown. The presence of trimeric helper proteins that may act as chaperones to coordinate export of type Vc ATs suggest simultaneous export and recent study points toward contemporaneous transport of all passenger polypeptides (Grin et al., 2014; Chauhan et al., 2019).

Likewise, the molecular mechanisms of many AT functions have still not been entirely elucidated.

In many cases of adhesins bind to a variety of surfaces promiscuously, but it is still not known as to whether different affinities toward different ECM molecules have a biological consequence. Furthermore, it is still not known how exactly differential binding works and which residues plays a role in differentiation of the binding targets. Another example for how ill-defined the functions of some passenger domains are protease and lipase targets. Although ATs with protease and lipase function have been studied for decades – and in fact belong to the best studied ATs – most of the host cellular targets have not been found yet. Even if targets have been defined in many cases the implications of targeting these structures is mostly unclear.

A lot of virulence potential lies within the secreted passengers of ATs, which would make many ATs potential targets for, e.g., drug and vaccine development (Xin et al., 2010; Olvera et al., 2011; Bentancor et al., 2012). Currently, there are already two major recombinant vaccines using ATs on the market. These contain FHA and Pertactin of *B. pertussis*, as well as NadA from *N. meningitidis* (Hellwig et al., 2003; Malito et al., 2014). Since ATs oftentimes belong to the virulence factors initiating an infection, for example Intimin and Invasin, one could also think of them as targets for anti-infective drugs (Durand et al., 2009; Heras et al., 2015).

In this review, we picked some prominent examples to illustrate the variety of passenger functions in ATs. Future research will undoubtedly lead to a more detailed picture of the variety of passenger functions and their involvement in infections as well as in symbiotic or environmental lifestyles.

## Author Contributions

IM wrote the first draft. All the authors contributed to complete the manuscript.

## Conflict of Interest Statement

The authors declare that the research was conducted in the absence of any commercial or financial relationships that could be construed as a potential conflict of interest.
